# Gut Microbiome as Target for Innovative Strategies Against Food Allergy

**DOI:** 10.3389/fimmu.2019.00191

**Published:** 2019-02-15

**Authors:** Roberto Berni Canani, Lorella Paparo, Rita Nocerino, Carmen Di Scala, Giusy Della Gatta, Ylenia Maddalena, Aniello Buono, Cristina Bruno, Luana Voto, Danilo Ercolini

**Affiliations:** ^1^Department of Translational Medical Science – Pediatric Section, University of Naples “Federico II”, Naples, Italy; ^2^European Laboratory for the Investigation of Food-Induced Diseases, University of Naples “Federico II”, Naples, Italy; ^3^ImmunoNutritionLab at CEINGE-Advanced Biotechnologies, University of Naples “Federico II”, Naples, Italy; ^4^Task Force on Microbiome Studies, University of Naples “Federico II”, Naples, Italy; ^5^Department of Agricultural Sciences, University of Naples “Federico II”, Naples, Italy

**Keywords:** immune tolerance, gut microbiota, mediterranean diet, dysbiosis, probiotics, gut microbiota metabolites, short chain fatty acids, butyrate

## Abstract

The dramatic increase in food allergy prevalence and severity globally requires effective strategies. Food allergy derives from a defect in immune tolerance mechanisms. Immune tolerance is modulated by gut microbiota function and structure, and microbiome alterations (dysbiosis) have a pivotal role in the development of food allergy. Environmental factors, including a low-fiber/high-fat diet, cesarean delivery, antiseptic agents, lack of breastfeeding, and drugs can induce gut microbiome dysbiosis, and have been associated with food allergy. New experimental tools and technologies have provided information regarding the role of metabolites generated from dietary nutrients and selected probiotic strains that could act on immune tolerance mechanisms. The mechanisms are multiple and still not completely defined. Increasing evidence has provided useful information on optimal bacterial species/strains, dosage, and timing for intervention. The increased knowledge of the crucial role played by nutrients and gut microbiota-derived metabolites is opening the way to a post-biotic approach in the stimulation of immune tolerance through epigenetic regulation. This review focused on the potential role of gut microbiome as the target for innovative strategies against food allergy.

## Introduction

### The Changing Scenario of Food Allergy

Food allergy (FA) is one of the most common allergic disorders in the pediatric age, and it has been considered as a global health problem, particularly in industrialized world (1). During the last two decades, studies have suggested that the epidemiology of FA has shown a dramatic increase in the prevalence, severity of clinical manifestations and risk of persistence into later ages, leading to an increase in medical visits, hospital admissions, treatments, burden of care on families, and economic impact, with an increase of costs for the families and healthcare system (2–4). According to the most recent epidemiological data, time trend analysis showed up to a 7-fold increase in hospital admissions for food severe allergic reactions in children in the UK, USA, Italy and Australia over the last 10 years (5–10). More than 170 foods have been identified as triggers of FA, such as tree nuts, eggs, peanuts, fish, shellfish, milk, wheat, soy, and seeds, with national and geographical variations concerning the most common FA (1, 10–15).

#### New Insights in the Pathogenesis of FA

**Figure 1:**
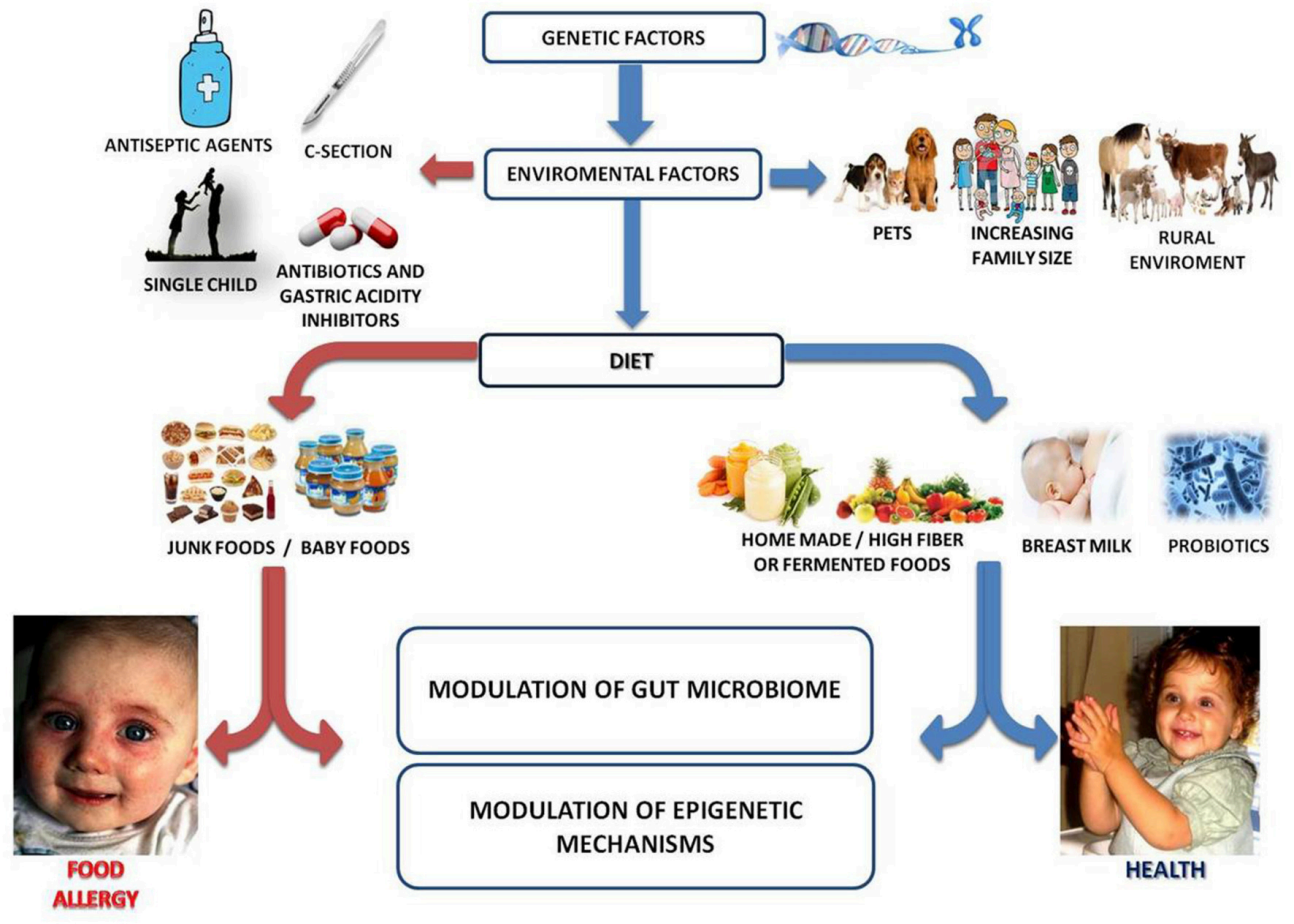
Gut microbiome as a target of intervention against food allergy. Several genetic, environmental, and dietary factors could modulate the gut microbiome-immune system axis influencing the occurrence of FA. For instance, increased family size, exposure to pets and/or rural environment, healthy diet (full of fibers, fermented foods, antioxidants, omega-3), breastfeeding and use of probiotics are associated with protection to FA. Conversely, C-section, prenatal, and early-life exposure to antibiotics/gastric acidity inhibitors/antiseptic agents, unhealthy diet (low fibers/high saturated fats and junk foods) may increase the risk for the development of FA. All these environmental factors act mainly on a modulation of gut microbiota structure and function which in turn could be responsible for the epigenetic regulation of genes involved in immune tolerance.

FA derives from a breakdown of immune tolerance to dietary antigens (16). Immune tolerance mechanisms involved the activation of dietary antigens specific regulatory T cell (Tregs) (17). Current knowledge suggests that the epidemiology of FA may be influenced by epigenome-genome-environment interactions leading to an alteration of immune system function (18, 19). To stabilize or fall the prevalence of FA, new and innovative strategies to reduce FA incidence are required. Many factors have been postulated to contribute to the onset of FA. The multiple immutable risk factors that could influence FA onset include male sex, ethnicity (increased risk among Asian and African Americans children), and genetics (familial risk, human leukocyte antigen (HLA), and specific genes) (2, 20–25). In addition, there are other modifiable factors that can be potentially targeted to reduce or prevent FA. These factors are related (mode of delivery, breast milk, use of antibiotics or gastric acidity inhibitors, use of antiseptic agents, rural environment, junk food-based and/or low-fiber/high-fat diet, consumption of unpasteurized milk or fermented foods, exposure to pets), or unrelated (comorbid atopic dermatitis, timing and route of exposure to foods, reduced consumption of omega-3-polyunsaturated fatty acids or vitamin D insufficiency, antioxidants,) to an influence on gut microbiome development and function (26–40) (Figure 1).

#### Clinical Consequences of Gut Microbiome Dysbiosis in Children With FA

**Figure 2:**
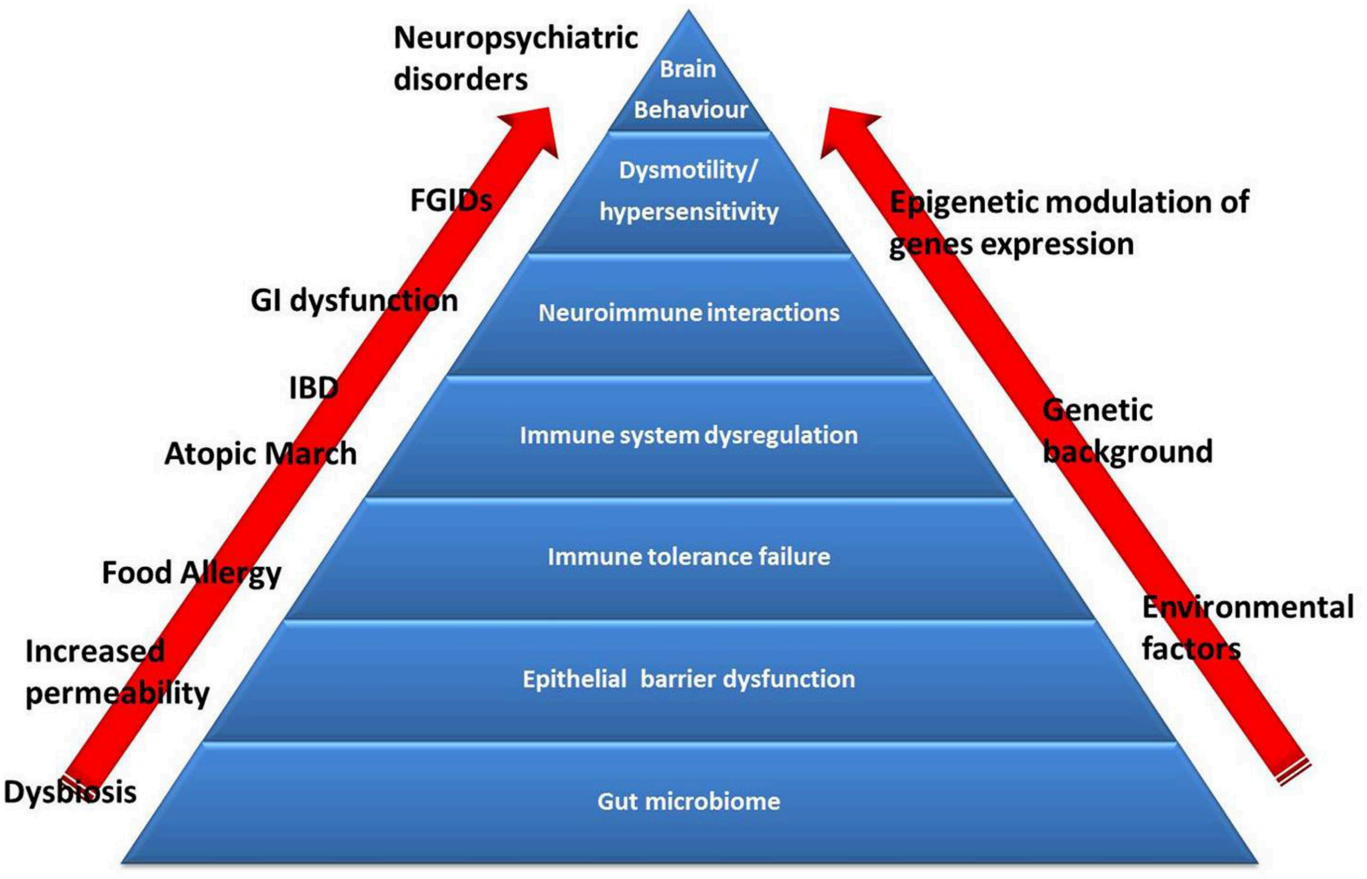
The Food Allergy pyramid. Children with FA present an increased risk to develop other conditions such as allergic disorders (atopic march), inflammatory bowel diseases (IBD), functional gastrointestinal disorders (FGIDs), and neuropsychiatric disorders. Several genetic factors are implicated in the pathogenesis of these conditions, but recent evidence suggest the pivotal role of gut microbiome dysbiosis (induced by environmental factors). Emerging evidence support the hypothesis of dysbiosis as the first hit in the development of alterations in intestinal barrier and immune system function (responsible for the occurrence of FA and atopic march) and dysregulation of the brain-gut endocrine-immune system axis (responsible for the occurrence of FGIDs, IBD, and neuropsychiatric disorders), at least in part through an activation of epigenetic mechanisms.

Many subjects with FA naturally outgrow it over time. Cow's milk allergy (CMA), hen's egg allergy and wheat allergy resolve in ~50% of children by the age of 5–10 years. Other FAs (including peanuts, tree nuts, fish) have low rates of resolution and are considered persistent (41). In addition, many forms of FA, may be related with later development of other allergic manifestations such as oculorhinitis, atopic dermatitis, asthma, and urticaria (the so called “Atopic March”) (42), as well as other diseases such as functional gastrointestinal disorders (FGIDs) (30, 43), inflammatory bowel diseases (IBD) (44), and psychiatric disorders, such as autistic spectrum disorders (ASD) attention deficit hyperactivity disorder (ADHD), and obsessive-compulsive disorder (OCD) (45). The pathogenesis of these events is still largely unknown, but increasing evidence suggest the hypothesis that a perturbation of gut microbiome, leading to alterations in immune system and gut-brain axis, could influence the occurrence of FA and FA-related conditions later in life (Figure 2).

### Gut Microbiome Features in FA

#### Investigating the Metagenomic and Metabolomics Features of Gut Microbiome

The knowledge and awareness of the roles played by gut microbiome and metabolites in the balance between health and disease is rapidly increasing. This is mainly due to advances in technology and the availability of high-sensitivity means to study microbial communities in any type of ecosystem. It is important for the clinicians and researchers dedicated to the FA field to know potential and limits of these technologies to better understand the value and significance of the findings reported in literature. Box 1 summarizes terminology for gut microbiota-based investigations in FA.

**Box 1:** A brief glossary for a better understanding of the potential of gut microbiota as target against food allergy.

Microbiota	The community of microbes in a particular ecosystem
Microbiome	The sum of micro-organisms, and their total genome capacity, in a particular environment
Operational taxonomic unit	A clusters of micro-organisms, grouped by DNA sequence similarity of a specific taxonomic marker gene. Operational taxonomic units are defined based on the similarity threshold (usually 97% similarity) set by the researcher
Microbiota diversity	A measure of how many different species are distributed in the community
Eubiosis	Healthy balance in a microbial ecosystem
Dysbiosis	A state of imbalance in a microbial ecosystem
Metagenomics	The study of the metagenome; the metagenome is the collective assembly of genomes from an environment (for example, the gut)
Metabolomics	The study of the metabolome; the metabolome is the collective array of metabolites present in a biological sample

Due to the power of genome DNA sequencing, we have learned much about the composition of gut microbial communities. In addition, the potential of transcriptomics, proteomics, and metabolomics are enlarging our understanding of the gut microbiota role in human health. Until the 1990s, knowledge of the gut microbiome was limited because the structure of gut microbiota was characterized using bacteriological culture. In the last decade, the composition of the gut microbiota was described by next generation sequencing of 16S ribosomal RNA genes. This is increasing the amount of information that can be retrieved by studying metagenomes from human samples, with the capability to infer the abundance of genes and potential metabolic pathways that characterize a microbial community. It is possible to describe the taxonomic composition of the microbiota and also to study the potential functions in a given system. Such methodological background is fundamental to investigate associations between microbiota structure and health as well as other environmental factors (46) and also to observe the changes of the gut microbiota in response to disease or perturbations in diet or lifestyle. An advanced technique to investigate gut microbiota at deep level is shotgun sequencing that represents a massive sequencing of the whole genome. Shotgun sequencing involves DNA random fragmentation, sequencing of these fragments and reconstruction of overlapping sequences to assemble them into a continuous sequence (47). Metabolomics represents one of the meta-omic approaches to study gut microbiota functions. Metabolomics uses high throughput techniques to characterize and quantify small molecules in several biofluids, such as feces, urine, plasma, serum, and saliva (48). The use of metabolomics is considered a powerful top-down systems biology approach, and it is essential to reveal the genetic-environment-health relationship, as well as the clinical biomarkers of diseases (49). Currently, the rapid development of several analytical platform, including liquid chromatography (LC), gas chromatography mass spectrometry (GC- MS), high-pressure LC (HPLC), ultra-pressure LC (UPLC), electrophoresis (CE) coupled to mass spectrometry (MS), Fourier transform infrared spectroscopy (FTIR), ion cyclotrone resonance-FT (ICR-FT), capillary and nuclear, and proton nuclear magnetic resonance spectroscopy (NMR-1H-NMR), allowed to better define bacteria related-metabolites and their metabolic pathways (50). Box 2 summarizes techniques used to investigate the gut microbiota metagenomic and metabolomic features. Gut microbiota metabolomic features are still largely unexplored. Metabolomics will provide important insides in the pathogenesis of FA. In this light, preliminary data available on short chain fatty acids (SCFA) profile are opening new perspective of intervention (see below). What is needed is a transition from descriptive research to understanding the ways the microbiome interacts with the host and plays a role in health and disease. In this frame, controlled clinical interventions are of utmost importance to establish microbiota causative involvement and are the basis to implement approaches of personalized medicine (51, 52). The study of the relationship between microbiome and FA may begin with association and be translated to causation and clinical practice with appropriate advances in knowledge. Wide screening of microbial diversity in gut microbiome of patients with a sure diagnosis of FA, including a well-matched control population, may identify useful signatures in the microbiome that are specific for certain types of FA (53). If the wide screening included cohorts of patients with different dietary style or ethnicity, the common microbial signatures would be even stronger and provide a solid indication of the microbial biomarkers of FA. Further mapping of the genomic features associated with FA may be inferred by metagenomics and metabolomics, which may provide information on the functional microbial signatures associated with FA.

**Box 2:** Techniques used to investigate the gut microbiota metagenomic and metabolomic features.

**Technique**	**Description**	**Advantages**	**Disadvantages**
*Metagenomics*			
Culture	Isolation of bacteria on selective media	Cheap, semi-quantitative	Labor intensive
qPCR	Amplification and quantification of 16S rRNA. Reaction mixture contains a compound that fluoresces when it binds to double-stranded DNA	Fast,quantitative, Phylogenetic identification	PCR bias, unable to identify unknown species
DGGE/TGGE	Gel separation of 16S rRNA amplicons using denaturant/ temperature	Fast, semi-quantitative, bands can be excised for further analysis	No phylogenetic identification, PCR bias
T-RFLP	Fluorescently labeled primers are amplified and then restriction enzymes are used to digest the 16S rRNA amplicon. Digested fragments separated by gel electrophoresis	Fast, cheap, semi-quantitative	No phylogenetic identification, PCR bias, low resolution
Fish	Fluorescently labeled oligonucleotide probes hybridize complementary target 16S rRNA sequences. When hybridization occurs, fluorescence can be enumerated using flow cytometry	Phylogenetic identification, semi-quantitative, no PCR bias	Dependent on probe sequences— unable to identify unknown species
DNA microarrays	Fluorescently labeled oligonucleotide probes hybridize with complementary nucleotide sequences. Fluorescence detected with a laser	Fast, Phylogenetic identification, semi-quantitative	Cross hybridization, PCR bias, species present in low levels can be difficult to detect
Cloned 16S rRNA gene sequencing	Cloning of full-length 16S rRNA amplicon, Sanger sequencing and capillary electrophoresis	Phylogenetic identification, quantitative	PCR bias, laborious, expensive, cloning bias
Direct sequencing of 16S rRNA amplicons	Massive parallel sequencing of partial 16S rRNA amplicons for example, 454 Pyrosequencing® (Roche Diagnostics GMBH Ltd, Mannheim, Germany) (amplicon immobilized on beads, amplified by emulsion PCR, addition of luciferase results in a chemoluminescent signal)	Fast, Phylogenetic identification, quantitative, identification of unknown bacteria	PCR bias, expensive, laborious
Microbiome shotgun sequencing	Massive parallel sequencing of the whole genome (e.g., 454 pyrosequencing® or Illumina®, San Diego, CA, USA)	Phylogenetic identification, quantitative	Expensive, analysis of data is computationally intense
*Metabolomics*			
Gas Chromatography Mass Spectrometry (GC-MS)	Thermally stable and volatile compounds are separated by GC and the eluting metabolites are detected by electron-impact (EI) mass spectrometers.	High efficiency, reproducibility and sensitivity	It can only be performed for volatile compounds
Liquid Chromatography Mass Spectrometry (LC)	Allows to separate compounds with little effort in a few pre-analytics steps (compared to GC-MS). The metabolite separation obtained with LC is followed by electro spray ionization (ESI) or atmospheric chemical ionization under pressure (APCI)	Lower temperatures of analysis, and it does not require sample volatility. Sensitivity, specificity, resolving power, and capability to extract additional information about metabolites from their retention time (RT) domain.	
Capillary Electrophoresis Mass Spectrometry (CE)	Offers high-analyte resolution and detect a wider spectrum of (polar) compounds compared to HPLC.	High resolution	It is properly applicable only to charged analytes
Fourier Transform Infrared Spectroscopy (FTIR)	Allows rapid, non-destructive and high-throughput determination of different sample types. This technique allows detecting different molecules, such as lipids and fatty acids (FAs), proteins, peptides, carbohydrates, polysaccharides, nucleic acids.	Ultra-high mass resolution able to distinguish slight variations in a wide number of mass signals, and allowing to obtain the structural identification of new biomarkers	Not high sensitivity and selectivity
Nuclear Magnetic Resonance Spectroscopy (NMR)	It uses the intramolecular magnetic field around atoms in molecules to change the resonance frequency, thus allowing access to details of molecules' electronic structure and obtaining information about their dynamics, reaction state, and chemical environment.	Useful to determine metabolic fingerprints leading to the identification and quantification of compounds in a non-targeted large-scale, in a non-destructive way, and with a high reproducibility	It is a relatively insensitive technique, and can only detect metabolites in high concentrations

Biomarker strains or defined microbial systems may be tested in gnotobiotic or humanized animal models to observe the development of the disease, and beneficial vs. detrimental microbial metabolites can be recognized and used as final targets of microbiome-targeted personalized interventions. The identification of bacterial metabolites that positively affect the immune tolerance network, may be an interesting strategy against FA using a post-biotic approach.

#### Evidence on Gut Microbiome Dysbiosis in FA

Mounting evidence indicates that gut microbiome dysbiosis early in life represents a critical factor underlying FA (26, 27, 54, 55). Experimental data from animal models suggest a link between gut microbiome and the occurrence of FA. Tregs was found reduced in mice treated with antibiotic or in *germ free* mice, with consequent predisposition to allergy development (56–58). Administration of defined Clostridia, or bacteria-derived short-chain fatty acids (SCFA) to *germ free* mice induced an increase of Treg cells number, and reduced allergic response (56, 59–62). The allergy-protective action of Clostridia was also confirmed in the animal model, where a significant protective effect consisting in regulation of innate lymphoid cell function, Foxp3^+^ Tregs, immunoglobulin (Ig)A and intestinal epithelial permeability was demonstrated (63). A “humanized mice model,” created with inoculation of microbiota-derived from human feces, resulted in an increase in Treg cells and a reduction of allergic symptoms (64). The functional role of dysbiosis associated with FA was also revealed by the different capacity of the gut microbiota of allergen-sensitized mice to increase Th2 cells number and IgE responses and to promote allergic sensitization (17).

Unfortunately, data characterizing the gut microbiome of patients affected by FA are still preliminary.

Table 1 summarizes main evidence on FA-associated gut microbiome features. Heterogeneity in study design, used to define the gut microbiome, make it difficult to establish a causal relationship between development of FA and specific bacteria. Despite these limitations, at least four relevant observations on FA-associated gut microbiome can be raised:

- Dysbiosis precedes the FA onset;
- Microbial community structure early in life, particularly in the first 6 months of life, is more relevant in FA development;
- No specific bacterial taxa could be consistently associated with FA onset, with a broad range of microbes that could have positive or negative influence on tolerogenic mechanisms;
- Dysbiosis could influence not only the occurrence, but also the disease course of FA. As suggested by different gut microbiota features comparing children who outgrow FA with patients with persistent form of FA (71).

**Table 1:** Main gut microbiome features in food allergy.

	**OTUs**	**Diversity**	**Technology**	**Main features**	**References**
Björkstén et al. (65) (*n* = 62; FA)	N.R.	N.R.	*Bacterial culture*	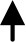 *Coliforms, S. Aureus* 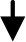 *Lactobacilli, Bifidobacteria*	(65)
Thompson-Chagoyan et al. (66) (*n* = 46:FA)	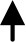	N.R.	*Bacterial culture*	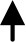 *Lactobacilli* 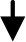 *Bifidobacteria*	(66)
Thompson-Chagoyan et al. (67) (*n* = 46:FA)	N.R.	N.R.	*Bacterial culture*	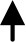 *C.coccoides, Atophium cluster*	(67)
Nakayama et al. (68) (*n* = 11: FA)	=	=	16s rRNA sequencing	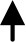 *Bacteroides,Propionibacterium*,*Klebsiella* 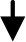 *Acinobacterium, Clostridium*	(68)
Ling et al. (69) (*n* = 34: FA)	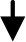	=	16s rRNA sequencing	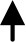 *Bacteroidetes, Proteobacteria*,*Actinobacteria* 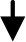 *Firmicutes*	(69)
Azad et al. (55) (*n* = 12: FS)	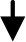	=	16s rRNA sequencing	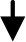 *Enterobacteriaceae*,*Bacteroidaceae*	(55)
Chen et al. (70) (*n* = 23: FS)	N.R.	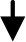	16s rRNA sequencing	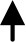 *Firmicutes*,*Proteobacteria*,*Actinobacteria* 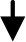 *Veillonella*	(70)
Berni Canani et al. (53) (*n* = 39; FA)	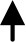	N.R.	16s rRNA sequencing	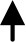 *Ruminococcaceae*,*Lachnospiraceae* 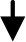 *Bifidobacteriaceae*,*Streptococcaceae*,*Enterobacteriaceae*	(53)
Bunyavanich et al. (71) (*n* = 226; FA)	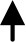	N.R.	16s rRNA sequencing	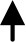 *Bacteroidetes, Enterobacter*	(71)
Inoue et al. (72) (*n* = 4: FA)	N.R.	N.R.	16s rRNA sequencing	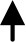 *Lachnospira, Veillionella*,*Suterella* 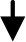 *Dorea, Akkermansia*	(72)
Kourosh et al. (73) (*n* = 68; FA)	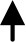	N.R.	16s rRNA sequencing	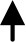 *Oscillobacter valericigenes*,*Lachnocrostidium bolteae*,*Faecalibacterium* sp.	(73)
Fazlollahi et al. (74) (*n* = 141; FA)	N.R.	N.R.	16s rRNA sequencing	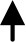 *Lachnospiraceae*,*Streptococcaceae*,*Leuconostocaceae*	(74)
Dong et al. (75) (*n* = 60; FA)	N.R.	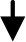	16s rRNA sequencing	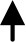 *Lactobacillaceae*, 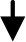 *Bifidobacteriaceae, Ruminococcaceae*	(75)
Berni Canani et al. (76) (*n* = 46; FA)	=	=	16s rRNA sequencing	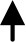 *Bacteroides, Alistipes*	(76)
Diaz et al. (77) (*n* = 27; FA)	N.R.	N.R.	16s rRNA sequencing	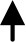 *Coriobacteriaceae*	(77)

*FA, food allergy; FS, sensitization to food antigens; OTUs, operational taxonomic units; N.R., not reported; 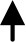, increase; 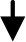, decrease*.

Recent studies underline the importance of the modulation of gut microbiota through different dietary interventions in pediatric patients with FA. CMA children treated with soy and rice based formula showed low fecal abundance of *Coriobacteriaceae* and *Bifidobacteriaceae*. Contrarily, *Coriobacteriaceae*, and certainly the genus Collinsella, the major bacteria that metabolized lactose in the gut, resulted increased in CMA children that consumed extensively hydrolyzed formula. In the same study, the authors found that fecal butyrate levels are positive correlated with abundance of *Coriobacteriaceae* (77). We showed that the treatment with extensively hydrolysed casein formula (EHCF) containing the probiotic *L. rhamnosus* GG (LGG) in CMA children significantly increased SCFA-producers bacteria and butyrate fecal levels. These effects were associated with immune tolerance acquisition (76).

### Targeting Gut Microbiome in FA

#### The Importance of the Diet-Gut Microbiome Axis

Advances in metagenomics and metabolomics implicate diet and gut microbiome (the diet-gut microbiome axis) as key modulators of the maturation of the immune system. Findings from a recent systematic review further support the relationship between maternal diet during pregnancy and lactation and FA during childhood (78). Diet from conception (maternal diet) up to the first 24 months of age (baby diet), may influence the risk of developing FA (78–81). A recent study suggests that a healthy diet with high levels of fruits, vegetables and home-made foods is associated with less FA at the age of 24 months (82). Several studies have reported that nutrients impact the gut microbiota and the production of bacterial metabolites (83, 84). The Mediterranean diet (MD) is defined as a healthy balanced diet. It is characterized by high consumption of assorted cereals, legumes, fruits, vegetables, olive oil, and nuts; moderate consumption of red wine, poultry and fish, and a lower intake of red meat and sweets. MD during pregnancy and early life has been demonstrated to have a protective role against allergic disease in children (85). These effects could derive from the high intake of non-digestible dietary carbohydrates (NDC), the beneficial fatty acid profile that is rich in omega-3, the high levels of polyphenols, and other antioxidants (86). Non-digestible dietary carbohydrates represent the primary nutrient source for gut bacteria, and their fermentation leads to the production of SCFAs) (53, 87). It has been demonstrated that reduced availability of NDC lowered the concentration of fiber-degrading bacteria and increased mucin-degrading bacteria (88). High adherence to the MD has been associated with-increased levels of *Prevotella* bacteria and other *Firmicutes* and of SCFAs production (89). The immunomodulatory mechanisms elicited by SCFAs represent one of the strongest connections between diet, gut microbiome and allergic diseases (90). Major SCFAs included acetate, propionate, butyrate, and valerate (87). SCFA-producing bacteria represent a functional group, including *Faecalibacterium prausnitzii* and *Eubacterium rectal, Roseburia* are efficient butyrate producers (91). SCFAs are major energy source for colonocytes and influence epigenetically several non-immune (tight junction proteins, mucus production) and immune functions (macrophages, neutrophils, dendritic cells (DCs), T and B cells) involved in the immune tolerance network (92–98). SCFAs interaction with enterocytes are mediated by G-protein coupled receptors, namely GPCRs; GPR41, GPR43, GPR109A, and Olfr78) (99–101). GPR43 and GPR41 are highly expressed by enterocytes (102), whereas immune cells express GPR43 and GPR109A (100, 103–106). Among SCFAs, butyrate exerts a pivotal role in immune tolerance induction. It has been found that SCFAs are able to increase colonic Treg frequency and *in vitro* treatment of colonic Tregs, from germ free mice, with propionate significantly increased *FoxP3* and IL-10 expression, a key cytokine that regulate Treg functions (60). Similarly, it has been demonstrated that butyrate facilitates generation of activated FoxP3^+^ Treg in mouse model (107).

Butyrate is able to regulate 103^+^DCs, reducing pro-inflammatory cytokines production and enhancing retinoic acid (RA) expression and subsequent generation of RA-regulated tolerogenic DCs (108). Butyrate promotes B cell differentiation and increases IgA and IgG production (107, 109).

The mechanisms are multiple and involve a strong epigenetic regulation of gene expression through the inhibition of histone deacetylase (HDAC) (60, 110, 111).

Butyrate deficiency has been observed in allergic children (112). Bacteria-produced SCFAs have been studies, has been specifically attributed to butyrate production by spore-forming Clostridiales. An enrichment of butyrate-producing taxa (Clostridia class and *Firmicutes phylum*) has been observed in children with faster CMA resolution (71). Altogether, these data suggest the potential of a “post-biotic” approach, based on the use of SCFAs against FA. In this light, data from our laboratory showed that oral butyrate induces a dramatic inhibition of acute allergic skin response, anaphylactic symptom score, body temperature decrease, intestinal permeability increase, anti-β lactoglobulin (BLG) IgE, IL-4, and IL-10 production in a murine model of CMA, suggesting a protective role of butyrate against FA (113).

We evaluated the direct effects of butyrate on peripheral blood mononuclear cells (PBMCs) from children affected by challenge-proven IgE-mediated CMA. PBMCs were stimulated with BLG in the presence or absence of butyrate. Preliminary results showed that butyrate stimulates IL-10 and IFN-γ production and decreases DNA methylation rate of these two cytokine genes. The same effective butyrate dose induces *FoxP3* demethylation and down-regulation of HDAC6/HDAC9 expression (113, 114). Additional potential mechanisms by which diet could exert pro-tolerogenic effects in the gut are related to the production of immunoregulatory metabolites, which interact with the host immune cells to promote non-responsiveness to innocuous luminal antigens (115). Tryptophan is an essential amino acid, which cannot be synthesized independently by humans; thus, it must be ingested through the diet. A portion of tryptophan is utilized to synthesize protein, and the other portion is catabolized to produce a variety of bioactive compounds, such as kynurenine (Kyn), serotonin and melatonin (84). Tryptophan absorbed by enterocytes directly activates the mTOR pathway by intracellular tryptophan receptors (116, 117). mTOR is known to play an important role in connecting metabolism and the immune system. During an inflammatory process, tryptophan is metabolized through the Kyn pathway. Kyn is an active metabolite and its biological activity is mediated by aryl hydrocarbon receptor (AhR) (118). The bond of Kyn to AhR receptor lead to the inhibition of DCs maturation and the proliferation of Th17 cells and Treg, increasing IL-22 and IL-10 production (119–122). Indole, indole 3-propionic acid (IPA) and indole-3-aldehyde (I3A) are produced by catabolism of tryptophan through intestinal commensal bacteria. A study demonstrated that strains of *Clostridium cadaveris* and *Peptostreptococcus anaerobius* CC14N metabolize tryptophan to produce IPA. Tryptophan can be also catabolized by lactobacilli to I3A. This metabolite protects gut mucosa against inflammation through AhR recognition (123). Indole-3-carbinole (I3C), an AhR ligand, has been demonstrated to boost immune tolerance in an ovalbumin (OVA)-sensitized mouse model (124). Mice fed I3C showed lower titres of anti-OVA IgG1 antibodies and higher number of CD103^+^MHC-II^+^ tolerogenic DCs compared to normal chow-fed control mice (124).

#### Engineering Gut Microbiome With Probiotics in FA

Immune tolerance is a major therapeutic target in FA. Evidence supports the concept that probiotics, defined as live microorganisms which when ingested in adequate amounts confer a beneficial effect on the host (125), could act at different levels in the immune tolerance network: modulating gut microbiota structure and function (increased production of butyrate) (53); interacting with enterocytes with subsequent modulation of non-immune (gut permeability and mucus thickness) (126–129) and immune tolerogenic mechanisms (stimulation of sIgA and β-defensins production) (130); modulation of cytokine response by immune cells (110–113, 131–134). Main pre-clinical evidence on probiotic activity against FA are summarized in Table 2. In the last decades, a number of experimental investigations have been developed to characterize organisms that could be used to modulate the immune system of patients with FA. Stimulation of human PBMCs with selected probiotic strains is a commonly used experimental tool for the investigation of the effect of these microorganisms on immune cells. The incubation of PBMCs with *L. plantarum* and *B. adolescentis* resulted in an increased production of the regulatory cytokine IL-10 by monocytes and DCs, and to enhanced IFN-γ production by T cells) (138, 148, 149). The addition of a probiotic mixture (*L. casei* W56, *L. lactis* W58, *L. acidophilus* W55, *L. salivarius* W57, *B. infantis* W52, *B. lactis* W18, and *B. longum* W51) to PBMCs from children with FA stimulated an increase of Th1 cells and related cytokines (141). An increase in T and B cells proliferation and a reduction in IgE production, were also observed in PBMCs from children with FA treated for 3 months with the same probiotic mixture (141). Using a 3D co-culture model of intestinal epithelial cells and PBMCs as an *in vitro* model of the intestinal mucosal immune system, Ghadimi et al. demonstrated that the probiotics *B. breve* and LGG inhibit activation of proinflammatory cytokines, IL-23, and IL-17, thereby reducing histone acetylation and simultaneously enhancing DNA methylation (135). The limitation of studying the effect of probiotics *in vitro* lies in the extrapolation of the results of *in vivo* benefits. For that reason, another commonly used experimental tool in this area is based on the use of animal model of FA. Using an OVA mouse model, it was demonstrated that oral administration of *B. infantis* reduced serum OVA-specific IgE, and IgG1 levels and Th2 cytokine release from splenocytes. Moreover, gut microbiota analysis showed that the probiotic-mediated protection was conferred by high abundance of *Coprococcus* and *Rikenella* (151). Different effects of oral administration of *B. coagulans* 09.712, *L. plantarum* 08.923, and *B. infantis* 11.322 in the reduction of Th2-driven intestinal inflammation and other symptoms associated with food-induced anaphylaxis, were demonstrated in a murine model of shrimp allergy (145). In particular, oral supplementation with *B. coagulans* 09.712 and *L. plantarum* 08.923 significantly ameliorates anaphylaxis symptoms and increases the population of CD4^+^ CD25^+^FoxP3^+^ T cells through mTORC inhibition, FoxP3 upregulation, and GATA-3 downregulation (145). Oral treatment with *C. butyricum* significantly ameliorated anaphylaxis symptoms and increased sIgA and FoxP3^+^Treg cells in the spleen from BLG-sensitized mice (150). Neonatal monocolonization of germ-free mice by *L. casei* BL2 modulated the allergic sensitization to cow's milk proteins, developed higher IgG responses against caseins, elicited by *L. casei* hydrolysed insoluble caseins into soluble immunogenic peptides (152). Similar results were obtained by others who observed a decrease of concentrations of IgE, IL-4, and IL-13 following administration of *B. infantis* CGMCC313-2 in BLG-sensitized mice (153). Oral administration of VSL#3 (a mixture of *Streptococcus thermophilus* BT01, *B. breve* BB02, *B. longum* BL03, *B. infantis* BI04, *L. acidophilus* BA05, *L. plantarum* BP06, *L. paracasei* BP07, *L. delbrueckii* subsp. *bulgaricus* BD08) to sensitized mice significantly reduces Th2 immune responses and protects against anaphylactic reactions in a mouse model of FA (154). Also, the incubation of mouse spleen cells from sensitized mice with probiotic mixture reduced allergen- stimulated IL-13 and IL-5 production and increased of IFN-γ and IL-10 production (154). An immunoregulatory action by LGG has been demonstrated in a murine model of CMA. LGG administration suppressed Th2 responses, such as reduced hypersensitivity score and lowered serum CMP-specific IgG1, while promoting IFN-γ and CMP-specific IgG2a levels (155). Similar results have been reported by our group in a BLG-sensitized mice model, in which we found that the administration of LGG added to EHCF elicited a significant reduction of allergic reaction, and of IL-4, IL-5, IL-13 and specific IgE production (139).

**Table 2:** Main preclinical evidences on the probiotics role against food allergy.

**Biological effects**	**Bacterial strains**	**References**
Intestinal barrier maturation	*B. lactis/bifidum*; *L. rhamnosus GG*	(128, 130, 135)
Th1/Th2 response balance: Th1 stimulation	*B. lactis*/*bifidum/ infantis*; *L. acidophilus/reuteri; L. rhamnosus GG*	(132, 133, 136, 137)
Th1/Th2 response balance: Th2 suppression	*B. bifidum/infantis/longum; L. actobacillus acidophilus/reuteri; L. rhamnosus GG*	(132, 134, 138–140)
Immune system regulation: Tregs development	*B. bifidum/infantis/lactis; L. acidophilus/reuteri/casei; L. rhamnosus GG*	(132, 134, 137)
Increase in B and T cell proliferation with enhanced production of Th1 and regulatory cytokines	*L. acidophilus; L. casei; L. salivarius; L. lactis; B. infantis; B. lactis; B. longum*	(135)
Immune system regulation: tolerogenic DCs development	*B. bifidum; L. reuteri/casei*; *L. rhamnosus GG*	(134, 137, 141, 142)
Suppression of IgE production	*B. bifidum/longum*; *B. lactis Bb-12*; *L. acidophilus*; *L. rhamnosus GG*	(128, 133, 138, 143, 144)
Epigenetic modulation of Th1/Th2 genes expression	*B. breve; L. rhamnosus GG*	(145–147)
Increase in the production of the regulatory cytokine IL-10 by monocytes and dendritic cells; enhance of IFN-γ production by T cells	*L. plantarum; B. adolescentis*	(141, 148, 149)
Increase in the population of CD4^+^FoxP3^+^ T cells, up-regulation of FoxP3 and down-regulation of GATA-3	*L. plantarum; B. coagulans*	(145)
Reduction of allergic reaction; reduction of IL-4, IL-5, IL-13 and specific IgE production	*L. rhamnosus GG*	(139)
Improvement of anaphylaxis symptoms and increase of sIgA and CD4^+^ CD25^+^ FoxP3Treg cell	*C. butyricum*	(150)

Clinical studies have investigated the efficacy of selected probiotic strains against FA. The effect appears strain-specific and more evident in the pediatric age group. In a randomized double-blind placebo-controlled trial, it was demonstrated that the administration of *L. casei* CRL431 and *B. lactis* BB12 added to hypoallergenic formula for 12 months did not modulate the rate of immune tolerance acquisition to cow's milk proteins in infants with CMA (140). Using a similar study design, we have demonstrated that EHCF containing the probiotic LGG is able to accelerate immune tolerance acquisition in CMA children. Children (aged 1–12 months), consecutively referred for suspected CMA (IgE- or non-IgE-mediated), but still receiving cow's milk proteins, were evaluated in the study. Subjects were randomly allocated to one of the two groups of dietary interventions: EHCF (control group); and EHCF containing LGG (at least 1.4 × 10^7^ CFU/100 mL; active group). After 12 months, the double-blind placebo- controlled food challenge was negative in 15 of 28 control infants (53.6%) and in 22 of 27 infants receiving EHCF with LGG [(81.5%, *p* = 0.027)] (156). The results were confirmed in a subsequent trial, when the effect of 5 different dietary strategies was investigated: EHCF, EHCF + LGG, partially hydrolyzed rice formula, soy formula or amino acid-based formula, in children affected by IgE- or non-IgE-mediated CMA. After the treatment period of 12 months, the proportion of children acquiring immune tolerance to cow's milk proteins was significantly higher in the group receiving EHCF+LGG (78.9%) than in other groups (157). At the 3-year follow- up of another pediatric cohort, a further confirmation of a greater rate of resolution of IgE-mediated CMA as well as a lower incidence of other atopic manifestations was described after treatment with EHCF+LGG (158). These effects could derive at least in part by a modulation elicited by selected LGG components on immune functions through different pathways including enterocytes, monocytes, mast cells, DCs and Tregs (159–162), and by an expansion of butyrate- producing gut microbiota (53, 76). Accordingly, studies in children with eczema and/or CMA who received EHCF plus LGG showed benefits in decreasing inflammation and gastrointestinal symptoms (163). Probiotics have been also proposed to reinforce the effectiveness of immunotherapy (164). Oral food immunotherapy (OIT) is currently the most investigated approach for persistent FA and it is based on the concept that repeated oral/intestinal exposures to antigens normally leads to tolerance. Randomized double-blind placebo- controlled trial was performed in 62 children with peanut allergy treated with fixed dose of probiotic together with peanut OIT (PPOIT) or placebo once daily for 18 months (165). Sustained unresponsiveness (SU), determined by double blind placebo controlled food challenge (DBPCFC), was achieved in 82.1% of children receiving PPOIT compared with 3.6% of those receiving placebo. PPOIT also induced high rates of resolution (90%) and was associated with reduced skin prick test reactivity, decreased peanut-specific IgE and increased peanut-specific IgG4 levels. No participants withdrawing because of adverse reactions.

No OIT control group was evaluated to determine if the probiotic itself had any effect on SU (165). Further studies are required to evaluate this approach comparing peanut OIT and probiotics with peanut OIT with placebo or probiotic alone.

## Conclusions

**Figure 3:**
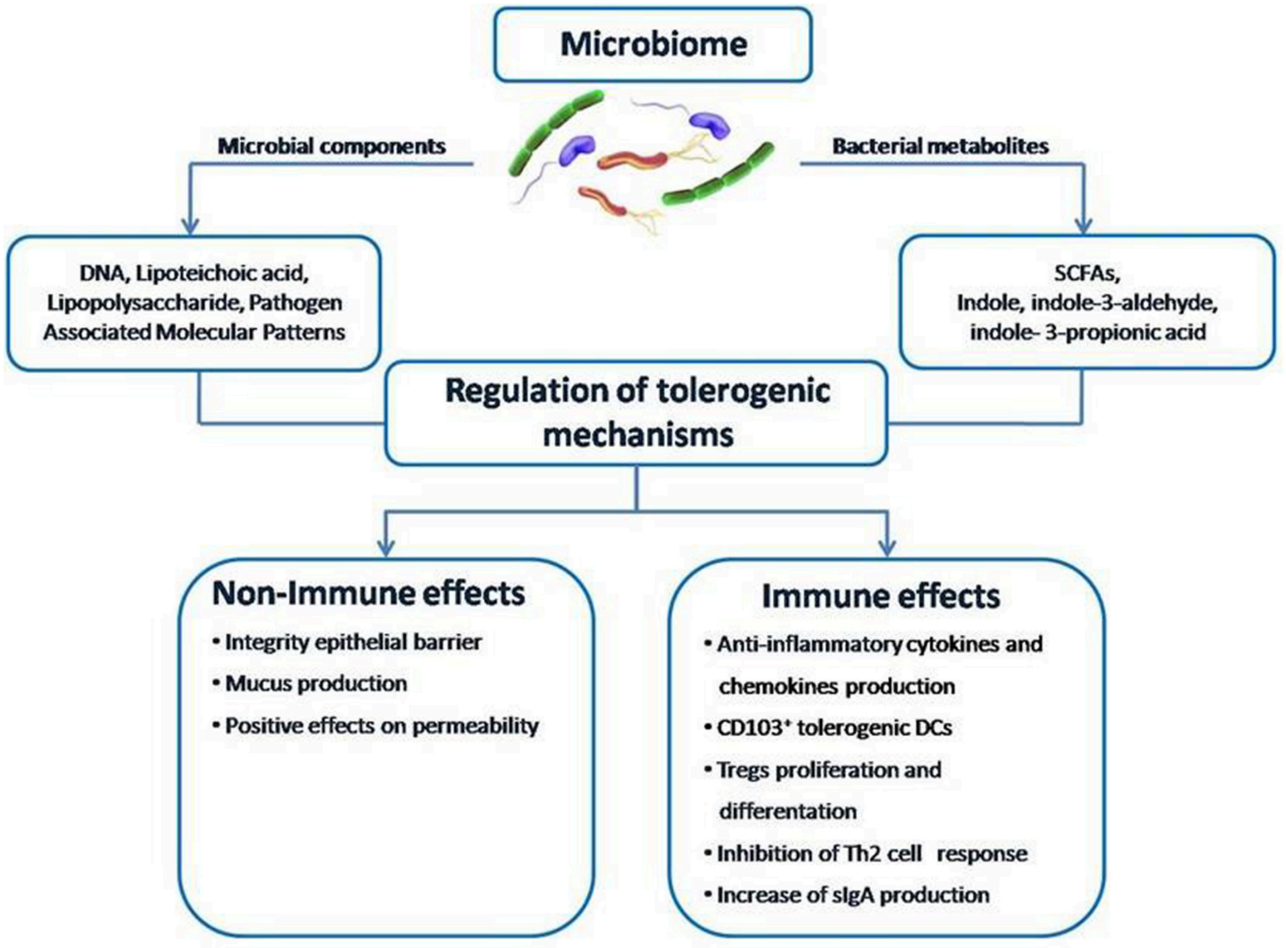
The structure of the gut microbiome-immune system axis. Within the gut microbiome-immune system axis the cross talk between microbes and the immune system may occur directly through microbial components or indirectly through the action of metabolites, such as SCFAs. A positive modulation of this axis can counteract the pathogenesis of FA by promoting epithelial integrity, gut permeability, mucus production, CD103^+^ tolerogenic DCs, Treg differentiation, cytokines production, and sIgA release from plasma cells.

Gut microbiome could be a promising target for innovative therapeutic and preventive strategies against FA. The results of the studies are encouraging, but more data are needed to better define the potential of modulating the diet-gut microbiome–immune system axis to fight FA (Figure 3). We are approaching a new era in which we can regulate immune system development and function through dietary intervention and measure the clinical impact through gut microbes and their metabolites. Given the current gaps in the investigational approaches and data analysis and interpretation, we need more scientific evidence that can be translated in clinical practice (Box 3).

**Box 3:** Targeting gut microbiota against FA: a research agenda.

**Targets**	**Possible strategies**
Identifying specific gut microbiota features associated with FA	To comparatively analyze metagenomics and metabolomics features of well-characterized populations of patients affected by different types of FA(naive of any dietary treatment) and healthy well-matched controls.
Characterizing the effect of dietary intervention and probiotic therapy	Prospective studies analyzing gut metagenomic and metabolomics changes in well-characterized populations.
Identifying the best probiotic strain to treat FA	Studies on mechanisms action in *in vitro* and in *in vivo* models. Clinical trials with well-characterized probiotic strains and doses involving patients with challenge-proven diagnosis of FA.
Optimizing the post-biotic approach to treat FA	Full characterization of the bio-functional features of gut microbiota metabolites that could be used against FA. Studies on mechanisms action in *in vitro* and in *in vivo* models. Clinical trials with well-characterized products involving patients with challenge-proven diagnosis of FA.

**Figure 4:**
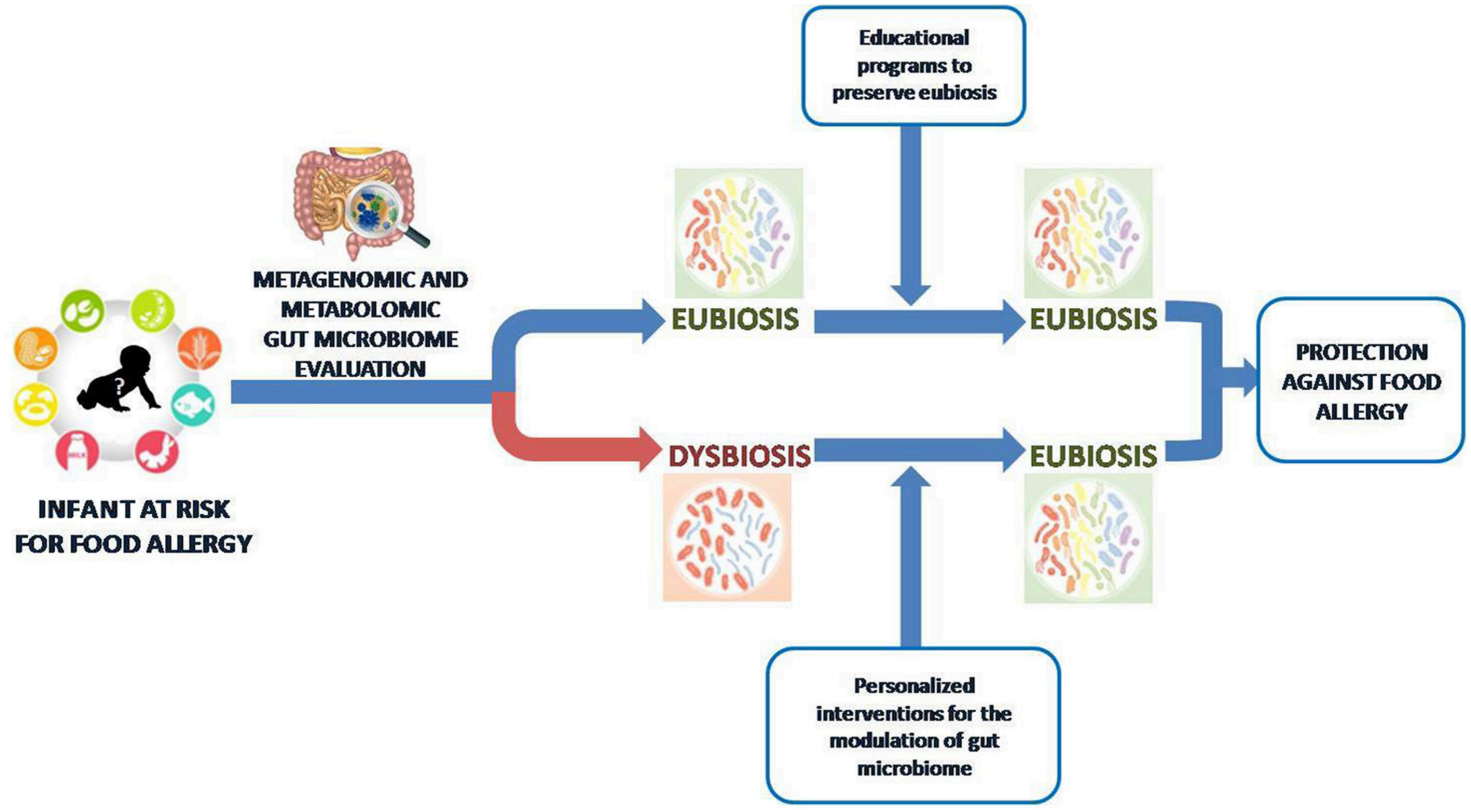
Toward a gut microbiome-based precision medicine against food allergy. We are approaching an era where the metagenomic and metabolomic evaluation of gut microbiota in children at risk for FA will drive personalized intervention to preserve or restore an “eubiosis” state based on nutritional counseling and educational programs.

Understanding how nutrients and metabolites, or probiotics could influence gut bacteria communities and the immune system will contribute to building up a precision medicine approach for FA care (Figure 4).

## Author Contributions

All authors listed have made a substantial, direct and intellectual contribution to the work, and approved it for publication.

### Conflict of Interest Statement

The authors declare that the research was conducted in the absence of any commercial or financial relationships that could be construed as a potential conflict of interest.

## Abbreviations

FA: food allergy
CMA: cow's milk allergy
EHCF: extensively hydrolyzed casein formula
LGG: *Lactobacillus rhamnosus* GG
OIT: oral food immunotherapy
SU: sustained unresponsiveness
PBMCs: peripheral blood mononuclear cells
BLG: β lactoglobulin
OVA: ovalbumin
LAB: lactic acid bacteria
NDC: Non-digestible dietary carbohydrates
SCFAs: short chain fatty acids
Tregs: regulatory T cells
DCs: dendritic cells
Kyn: kynurenine
AhR: arylhydrocarbonreceptor
IPA: indole 3-propionic acid
I3A: indole-3-aldehyde
I3C: indole-3-carbinole.

## References

[1] Boyce JA Assa'ad A Burks AW Assa'ad A Burks AW Jones SM Guidelines for the diagnosis and management of food allergy in the United States: report of the NIAID–sponsored expert panel. J Allergy Clin Immunology (2010) 126:1105–18. 10.1016/j.jaci.2010.10.007 21134568 PMC4241958

[2] Loh W Tang MLK. The epidemiology of food allergy in the global context. Int J Environ Res Public Health (2018) 15:2043. 10.3390/ijerph15092043 30231558 PMC6163515

[3] McBride D Keil T Grabenhenrich L Dubakiene R Drasutiene G Fiocchi A. The EuroPrevall birth cohort study on food allergy: baseline characteristics of 12,000 newborns and their families from nine European countries. Pediatr Allergy Immunol. (2012) 23:230–9. 10.1111/j.1399-3038.2011.01254.x 22192443

[4] Leung ASY Wong GWK Tang MLK. Food allergy in the developing world. J Allergy Clin Immunol. (2018) 141:76–8. 10.1016/j.jaci.2017.11.008 29174528

[5] Berni Canani R Nocerino R Terrin G Leone L Troncone R. Hospital admissions for food–induced anaphylaxis in Italian children. Clin Exp Allergy (2012) 42:1813–4. 10.1111/cea.12036 23181797

[6] Turner PJ Gowland MH Sharma V Ierodiakonou D Harper N Garcez T. Increase in anaphylaxis–related hospitalizations but no increase in fatalities: an analysis of United Kingdom national anaphylaxis data, 1992–2012. J Allergy Clin Immunol. (2015) 135:956–63.e1. 10.1016/j.jaci.2014.10.021 25468198 PMC4382330

[7] Mullins RJ Dear KB Tang ML. Time trends in Australian hospital anaphylaxis admissions in 1998–1999 to 2011–2012. J Allergy Clin Immunol. (2015) 136:367–75. 10.1016/j.jaci.2015.05.009 26187235

[8] Nocerino R Leone L Cosenza L Berni Canani R. Increasing rate of hospitalizations for food–induced anaphylaxis in Italian children: an analysis of the Italian Ministry of Health database. J Allergy Clin Immunol. (2015) 135:833–5. 10.1016/j.jaci.2014.12.1912 25630939

[9] Mullins RJ Wainstein BK Barnes EH Liew WK Campbell DE. Increases in anaphylaxis fatalities in Australia from 1997 to 2013. Clin Exp Allergy (2016) 46:1099–110. 10.1111/cea.12748 27144664

[10] Sicherer SH Muñoz–Furlong A Godbold JH Sampson HA. US prevalence of self–reported peanut, tree nut, and sesame allergy: 11–year follow–up. J Allergy Clin Immunol. (2010) 125:1322–6. 10.1016/j.jaci.2010.03.029 20462634

[11] Ben–Shoshan M Harrington DW Soller L Fragapane J Joseph L St Pierre Y. A population–based study on peanut, tree nut, fish, shellfish, and sesame allergy prevalence in Canada. J Allergy Clin Immunol. (2010) 125:1327–35. 10.1016/j.jaci.2010.03.015 20451985

[12] Chafen JJ Newberry SJ Riedl MA Bravata DM Maglione M Suttorp MJ. Diagnosing and managing common food allergies: a systematic review. JAMA (2010) 303:1848–56. 10.1001/jama.2010.582 20460624

[13] Osborne NJ Koplin JJ Martin PE Gurrin LC Lowe AJ Matheson MC. Prevalence of challenge–proven IgE–mediated food allergy using population–based sampling and predetermined challenge criteria in infants. J Allergy Clin Immunol. (2011) 127:668–76.e2. 10.1016/j.jaci.2011.01.039 21377036

[14] Gupta RS Springston EE Warrier MR Smith B Kumar R Pongracic J. The prevalence, severity, and distribution of childhood food allergy in the United States. Pediatrics (2011) 128:e9–17. 10.1542/peds.2011-0204 21690110

[15] National Academies of Sciences Engineering and Medicine Finding a Path to Safety in Food Allergy: Assessment of Global Burden, Causes, Prevention, Management, and Public Policy. Washington, DC: National Academies Press (2016). 28609025

[16] Sicherer SH Sampson HA. Food allergy: a review and update on epidemiology, pathogenesis, diagnosis, prevention, and management. J Allergy Clin Immunol. (2018) 141:41–58. 10.1016/j.jaci.2017.11.003 29157945

[17] Noval Rivas M Burton OT Wise P Zhang YQ Hobson SA Garcia Lloret M. A microbiota signature associated with experimental food allergy promotes allergic sensitization and anaphylaxis. J Allergy Clin Immunol. (2013) 131:201–12. 10.1016/j.jaci.2012.10.026 23201093 PMC3860814

[18] Berni Canani R Gilbert JA Nagler CR. The role of the commensal microbiota in the regulation of tolerance to dietary allergens. Curr Opin Allergy Clin Immunol. (2015) 15:243–9. 10.1097/ACI.0000000000000157 25827065 PMC4498960

[19] Paparo L Aitoro R Nocerino R di Scala C Di Costanzo M Cosenza L Epigenetic regulation of early nutrition on immune system. In: Preedy VR Patel VB, editors. Handbook of Nutrition, Diet, and Epigenetics Cham: Springer International Publishing AG (2018). 10.1007/978-3-319-31143-2_54-1

[20] Peters RL Allen KJ Dharmage SC Lodge CJ Koplin JJ Ponsonby AL. Differential factors associated with challenge–proven food allergy phenotypes in a population cohort of infants: a latent class analysis. Clin Exp Allergy (2015) 45:953–63. 10.1111/cea.12478 25523199

[21] Sicherer SH Mu noz–Furlong A Sampson HA Prevalence of sea food allergy in the United States determined by a random telephone survey. J Allergy Clin Immunol. (2004) 114:159–65. 10.1016/j.jaci.2004.04.018 15241360

[22] Panjari M Koplin JJ Dharmage SC Peters RL Gurrin LC Sawyer SM. Nut allergy prevalence and differences between Asian born children and Australian born children of Asian descent: a state–wide survey of children at primary school entry in Victoria, Australia. Clin Exp Allergy (2016) 46:602–9. 10.1111/cea.12699 26728850

[23] Sicherer SH Wood RA Vickery BP Jones SM Liu AH Fleischer DM. The natural history of egg allergy in an observational cohort. J Allergy Clin Immunol. (2014) 133:492–9. 10.1016/j.jaci.2013.12.1041 24636473 PMC3959659

[24] Hourihane JO Dean TP Warner JO. Peanut allergy in relation to heredity, maternal diet, and other atopic diseases: results of a questionnaire survey, skin prick testing, and food challenges. BMJ (1996) 313:518–2. 10.1136/bmj.313.7056.518 8789975 PMC2351952

[25] Lichtenstein P Svartengren M. Genes, environments, and sex: factors of importance in atopic diseases in 7–9–year–old Swedish twins. Allergy (1997) 52:1079–86. 10.1111/j.1398-9995.1997.tb00179.x 9404559

[26] Savage JH Lee–Sarwar KA Sordillo J Bunyavanich S Zhou Y O'Connor G. A prospective microbiome wide association study of food sensitization and food allergy in early childhood. Allergy (2018) 73:145–52. 10.1111/all.13232 28632934 PMC5921051

[27] Prince BT Mandel MJ Nadeau K Singh AM. Gut microbiome and the development of food allergy and allergic disease. Pediatr Clin North Am. (2015) 62:1479–92. 10.1016/j.pcl.2015.07.007 26456445 PMC4721650

[28] Mitselou N Hallberg J Stephansson O Almqvist C Melén E Ludvigsson JF. Cesarean delivery, preterm birth, and risk of food allergy: Nationwide Swedish cohort study of more than 1 million children. J Allergy Clin Immunol. (2018) 142:1510–14. 10.1016/j.jaci.2018.06.044 30213656

[29] Du Toit G Roberts G Sayre PH Plaut M Bahnson HT Mitchell H. Identifying infants at high risk of peanut allergy: the Learning Early About Peanut Allergy (LEAP) screening study. J Allergy Clin Immunol. (2013) 131:135–43.e1–12. 10.1016/j.jaci.2012.09.015 23174658

[30] Biasucci G Rubini M Riboni S Morelli L Bessi E Retetangos C. Mode of delivery affects the bacterial community in the newborn gut. Early Hum Dev. (2010) 86(Suppl 1):13–5. 10.1016/j.earlhumdev.2010.01.004 20133091

[31] Guibas GV Moschonis G Xepapadaki P Roumpedaki E Androutsos O Manios Y. Conception via in vitro fertilization and delivery by Caesarean section are associated with paediatric asthma incidence. Clin Exp Allergy (2013) 43:1058–66. 10.1111/cea.12152 23957341

[32] Greenwood C Morrow AL Lagomarcino AJ Altaye M Taft DH Yu Z. Early empiric antibiotic use in preterm infants is associated with lower bacterial diversity and higher relative abundance of Enterobacter. J Pediatr. (2014) 165:23–9. 10.1016/j.jpeds.2014.01.010 24529620 PMC4074569

[33] Arboleya S Sánchez B Milani C Duranti S Solís G Fernández N. Intestinal microbiota development in preterm neonates and effect of perinatal antibiotics. J Pediatric. (2015) 166:538–44. 10.1016/j.jpeds.2014.09.041 25444008

[34] Fouhy F Guinane CM Hussey S Wall R Ryan CA Dempsey EM. High–throughput sequencing reveals the incomplete, short–term recovery of infant gut microbiota following parenteral antibiotic treatment with ampicillin and gentamicin. Antimicrob Agents Chemother. (2012) 56:5811–20. 10.1128/AAC.00789-12 22948872 PMC3486619

[35] Tanaka S Kobayashi T Songjinda P Tateyama A Tsubouchi M Kiyohara C. Influence of antibiotic exposure in the early postnatal period on the development of intestinal microbiota. FEMS Immunol Med Microbiol. (2009) 56:80–7. 10.1111/j.1574-695X.2009.00553.x 19385995

[36] Kummeling I Stelma FF Dagnelie PC Snijders BE Penders J Huber M. Early life exposure to antibiotics and the subsequent development of eczema, wheeze, and allergic sensitization in the first 2 years of life: the KOALA Birth Cohort Study. Pediatrics (2007) 119:e225–31. 10.1542/peds.2006-0896 17200248

[37] Zivkovic AM German JB Lebrilla CB Mills DA. Human milk glycobiome and its impact on the infant gastrointestinal microbiota. Proc Natl Acad Sci USA. (2011) 108(Suppl 1):4653–8. 10.1073/pnas.1000083107 20679197 PMC3063602

[38] Silvers KM Frampton CM Wickens K Pattemore PK Ingham T Fishwick D. Breastfeeding protects against current asthma up to 6 years of age. J Pediatr. (2012) 160:991–6. 10.1016/j.jpeds.2011.11.055 22289356

[39] Wu GD Chen J Hoffmann C Bittinger K Chen YY Keilbaugh SA. Linking long–term dietary patterns with gut microbial enterotypes. Science (2011) 334:105–8. 10.1126/science.1208344 21885731 PMC3368382

[40] Trompette A Gollwitzer ES Yadava K Sichelstiel AK Sprenger N Ngom–Bru C. Gut microbiota metabolism of dietary fiber influences allergic airway disease and hematopoiesis. Nat Med. (2014) 20:159–66. 10.1038/nm.3444 24390308

[41] Savage J Sicherer S Wood R. The natural history of food allergy. J Allergy Clin Immunol Pract. (2016) 4:196–203. 10.1016/j.jaip.2015.11.024 26968958

[42] Hill DA Spergel JM. The atopic march: critical evidence and clinical relevance. Ann Allergy Asthma Immunol. (2018) 120:131–7. 10.1016/j.anai.2017.10.037 29413336 PMC5806141

[43] Saps M Lu P Bonilla S. Cow's–milk allergy is a risk factor for the development of FGIDs in children. J Pediatr Gastroenterol Nutr. (2011) 52:166–9. 10.1097/MPG.0b013e3181e85b55 20975580

[44] Virta LJ Kautiainen H Kolho KL. Symptoms suggestive of cow's milk allergy in infancy and pediatric inflammatory bowel disease. Pediatr Allergy Immunol. (2016) 27:361–7. 10.1111/pai.12551 26887931

[45] Topal E Catal F Soylu N Ozcan OO Celiksoy MH Babayigit A. Psychiatric disorders and symptoms severity in pre–school children with cow's milk allergy. Allergol Immunopathol. (2016) 44:445–9. 10.1016/j.aller.2016.03.001 27240441

[46] Gilbert JA Blaser MJ Caporaso JG Jansson JK Lynch SV Knight R. Current understanding of the human microbiome. Nat Med. (2018) 24:392–400. 10.1038/nm.4517 29634682 PMC7043356

[47] Fraher MH O'Toole PW Quigley EM. Techniques used to characterize the gut microbiota: a guide for the clinician. Nat Rev Gastroenterol Hepatol. (2012) 9:312–2. 10.1038/nrgastro.2012.44 22450307

[48] Nicholson JK Lindon JC Systems biology: metabolomics. Nature (2008) 455:1054–6. 10.1038/4551054a 18948945

[49] Moco S Collino S Rezzi S Martin FP. Metabolomics perspectives in pediatric research. Pediatr Res. (2013) 73:570–6. 10.1038/pr.2013.1 23314292

[50] Vernocchi P Del Chierico F Putignani L. Gut microbiota profiling: metabolomics based approach to unravel compounds affecting human health. Front Microbiol. (2016) 7:1144. 10.3389/fmicb.2016.01144 27507964 PMC4960240

[51] De Filippis F Vitaglione P Cuomo R Berni Canani R Ercolini D. Dietary interventions to modulate the gut microbiome—how far away are we from precision medicine. Inflamm Bowel Dis. (2018) 24:2142–54. 10.1093/ibd/izy080 29668914

[52] Bashiardes S Godneva A Elinav E Segal E Bashiardes S Godneva A Elinav E. Towards utilization of the human genome and microbiome for personalized nutrition. Curr Opin Biotechnol. (2018) 51:57–63. 10.1016/j.copbio.2017.11.013 29223004

[53] Berni Canani R Sangwan N Stefka AT Nocerino R Paparo L Aitoro R. Lactobacillus rhamnosus GG supplemented formula expands butyrate producing bacterial strains in food allergic infants. ISME J. (2016) 10:742–50. 10.1038/ismej.2015.151 26394008 PMC4817673

[54] Fieten KB Totté JEE Levin E Reyman M Meijer Y Knulst A. Fecal microbiome and food allergy in pediatric atopic dermatitis: a cross–sectional pilot study. Clin Allergol. (2018) 175:77–84. 10.1159/000484897 29393195

[55] Azad MB Konya T Guttman DS Field CJ Sears MR HayGlass KT. Infant gut microbiota and food sensitization: associations in the first year of life. Clin Exp Allergy (2015) 45:632–43. 10.1111/cea.12487 25599982

[56] Atarashi K Tanoue T Shima T Imaoka A Kuwahara T Momose Y. Induction of colonic regulatory T cells by indigenous Clostridium species. Science (2011) 331:337–41. 10.1126/science.1198469 21205640 PMC3969237

[57] Russell SL Gold MJ Hartmann M Willing BP Thorson L Wlodarska M. Early life antibiotic–driven changes in microbiota enhance susceptibility to allergic asthma. EMBO Rep. (2012) 13:440–7. 10.1038/embor.2012.32 22422004 PMC3343350

[58] Bashir ME Louie S Shi HN Nagler–Anderson C. Toll–like receptor 4 signaling by intestinal microbes influences susceptibility to food allergy. J Immunol. (2004) 172:6978–87. 10.4049/jimmunol.172.11.6978 15153518

[59] Geuking MB Cahenzli J Lawson MA Ng DC Slack E Hapfelmeier S. Intestinal bacterial colonization induces mutualistic regulatory T cell responses. Immunity (2011) 34:794–806. 10.1016/j.immuni.2011.03.021 21596591

[60] Smith PM Howitt MR Panikov N Michaud M Gallini CA Bohlooly–Y M. The microbial metabolites, short chain fatty acids, regulate colonic Treg cell homeostasis. Science (2013) 341:569–73. 10.1126/science.1241165 23828891 PMC3807819

[61] Lathrop SK Bloom SM Rao SM Nutsch K Lio CW Santacruz N. Peripheral education of the immune system by colonic commensal microbiota. Nature (2011) 478:250–4. 10.1038/nature10434 21937990 PMC3192908

[62] Mazmanian SK Round JL Kasper DL. A microbial symbiosis factor prevents intestinal inflammatory disease. Nature (2008) 453:620–5. 10.1038/nature07008 18509436

[63] Stefka AT Feehley T Tripathi P Qiu J McCoy K Mazmanian SK. Commensal bacteria protect against food allergen sensitization. PNAS (2014) 111:13145–50. 10.1073/pnas.1412008111 25157157 PMC4246970

[64] Atarashi K Tanoue T Oshima K Suda W Nagano Y Nishikawa H. Treg induction by a rationally selected mixture of Clostridia strains from the human microbiota. Nature (2013) 500:232–6. 10.1038/nature12331 23842501

[65] Bjorksten B Naaber P Sepp E Mikelsaar M. The intestinal microflora in allergic Estonian and Swedish 2–year–old children. Clin Exp Allergy (1999) 29:342–6. 10202341 10.1046/j.1365-2222.1999.00560.x

[66] Thompson–Chagoyan OC Vieites JM Maldonado J Edwards C Gil A. Changes in faecal microbiota of infants with cow's milk protein allergy – a Spanish prospective case–control 6–month follow–up study. Pediatr Allergy Immunol. (2010) 21:e394–e400. 10.1111/j.1399-3038.2009.00961.x 19889194

[67] Thompson–Chagoyan OC Fallani M Maldonado J Vieites JM Khanna S Edwards C. Faecal microbiota and short–chain fatty acid levels in faeces from infants with cow‘s milk protein allergy. Int Arch Allergy Immunol. (2011) 156:325–32. 10.1159/000323893 21720179

[68] Nakayama J Kobayashi T Tanaka S Korenori Y Tateyama A Sakamoto N. Aberrant structures of fecal bacterial community in allergic Infants profiled by 16S rRNA gene pyrosequencing. FEMS Immunol Med Microbiol. (2011) 63:397–406. 10.1111/j.1574-695X.2011.00872.x 22029688

[69] Ling Z Li Z Liu X Cheng Y Luo Y Tong X. Altered fecal microbiota composition associated with food allergy in infants. App Environ Microbiol. (2014) 80:2546–54. 10.1128/AEM.00003-14 24532064 PMC3993190

[70] Chen CC Chen KJ Kong MS Chang HJ Huang JL Alterations in the gut microbiota of children with food sensitization in early life. Pediatr Allergy Immunol. (2016) 27:254–62. 10.1111/pai.12522 26663491

[71] Bunyavanich S Shen N Grishin A Wood R Burks W Dawson P. Early–life gut microbiome composition and milk allergy resolution. J Allergy Clin Immunol. (2016) 138:1122–30. 10.1016/j.jaci.2016.03.041 27292825 PMC5056801

[72] Inoue R Sawai T Sawai C Nakatani M Romero–Pérez GA Ozeki M. A preliminary study of gut dysbiosis in children with food allergy. Biosci Biotechnol Biochem. (2017) 81:2396–9. 10.1080/09168451.2017.1383849 29017394

[73] Kourosh A Luna RA Balderas M Nance C Anagnostou A Devaraj S. Fecal microbiome signatures are different in food– allergic children compared to siblings and healthy children. Pediatr Allergy Immunol. (2018) 29:545–54. 10.1111/pai.12904 29624747

[74] Fazlollahi M Chun Y Grishin A Wood RA Burks AW Dawson P. Early–life gut microbiome and egg allergy. Allergy (2018) 73:1515–24. 10.1111/all.13389 29318631 PMC6436531

[75] Dong P Feng JJ Yan DY Lyu YJ Xu X. Early–life gut microbiome and cow's milk allergy– a prospective case – control 6–month follow–up study. Saudi J Biol Sci. (2018) 25:875–80. 10.1016/j.sjbs.2017.11.051 30108435 PMC6088111

[76] Berni Canani R De Filippis F Nocerino R Paparo L Di Scala C Cosenza L. Gut microbiota composition and butyrate production in children affected by non–IgE–mediated cow's milk allergy. Sci Rep. (2018) 8:12500. 10.1038/s41598-018-30428-3 30131575 PMC6104073

[77] Díaz M Guadamuro L Espinosa–Martos I Mancabelli L Jiménez S Molinos–Norniella C. Microbiota and derived parameters in fecal samples of infants with non–IgE Cow's milk protein allergy under a restricted diet. Nutrients (2018) 10:1481. 10.3390/nu10101481 30314304 PMC6213916

[78] Garcia–Larsen V Ierodiakonou D Jarrold K Cunha S Chivinge J Robinson Z Diet during pregnancy and infancy risk of allergic or autoimmune disease: a systematic review and meta–analysis. PLoS Med. (2018) 15:e1002507 10.1371/journal.pmed.1002507 29489823 PMC5830033

[79] Barker DJP. Developmental origins of chronic disease. Public Health (2012) 126:185–9. 10.1016/j.puhe.2011.11.014 22325676

[80] Netting MJ Middleton PF Makrides M Does maternal diet during pregnancy and lactation affects outcomes in offspring? A systematic review of food–based approaches. Nutrition (2014) 30:1225–41. 10.1016/j.nut.2014.02.015 25280403

[81] Wopereis H Oozeer R Knipping K Belzer C Knol J. The first thousand days – intestinal microbiology of early life: establishing a symbiosis. Pediatr Allergy Immunol. (2014) 25:428–38. 10.1111/pai.12232 24899389

[82] Grimshaw KE Maskell J Oliver EM Morris RC Foote KD Mills EN. Diet and food allergy development during infancy: birth cohort study findings using prospective food diary data. J Allergy Clin Immunol. (2014) 133:511–9. 10.1016/j.jaci.2013.05.035 23891269

[83] Rooks MG Garrett WS. Gut microbiota, metabolites and host immunity. Nat Rev Immunol. (2016) 16:341–52. 10.1038/nri.2016.42 27231050 PMC5541232

[84] Ma N Guo P Zhang J He T Kim SW Zhang G Nutrients mediate intestinal bacteria–mucosal immune cross talk. Front Immunol. (2018) 9:5 10.3389/fimmu.2018.00005 29416535 PMC5787545

[85] Castro–Rodriguez JA Garcia–Marcos L. What are the effects of a Mediterranean diet on allergies and asthma in children? Front Pediatr. (2017) 5:72. 10.3389/fped.2017.00072 28484688 PMC5399020

[86] Castro–Rodriguez JA Ramirez–Hernandez M Padilla O Pacheco–Gonzalez RM Pérez–Fernández V Garcia–Marcos L. Effect of foods and Mediterranean diet during pregnancy and first years of life on wheezing, rhinitis and dermatitis in preschoolers. Allergol Immunopathol. (2016) 44:400–9. 10.1016/j.aller.2015.12.002 27087566

[87] Berni Canani R Costanzo MD Leone L Pedata M Meli R Calignano A Potential beneficial effects of butyrate in intestinal and extraintestinal diseases. World J Gastroenterol. (2011) 17:1519–28. 10.3748/wjg.v17.i12.1519 21472114 PMC3070119

[88] Desai MS Seekatz AM Koropatkin NM Kamada N Hickey CA Wolter M. A dietary fiber–deprived gut microbiota degrades the colonic mucus barrier and enhances pathogen susceptibility. Cell (2016) 167:1339. 10.1016/j.cell.2016.10.043 27863247 PMC5131798

[89] De Filippis F Pellegrini N Vannini L Jeffery IB La Storia A Laghi L. High–level adherence to a Mediterranean diet beneficially impacts the gut microbiota and associated metabolome. Gut (2016) 65:1812–21. 10.1136/gutjnl-2015-309957 26416813

[90] McKenzie C Tan J Macia L Mackay CR. The nutrition–gut microbiome–physiology axis and allergic diseases. Immunol Rev. (2017) 278:277–95. 10.1111/imr.12556 28658542

[91] Louis P Flint HJ. Diversity, metabolism and microbial ecology of butyrate–producing bacteria from the human large intestine. FEMS Microbiol Lett. (2009) 294:1–8. 10.1111/j.1574-6968.2009.01514.x 19222573

[92] Schauber J Svanholm C Termen S Iffland K Menzel T Scheppach W Expression of the cathelicidin LL−37 is modulated by short chain fatty acids in colonocytes: relevance of signaling pathways. Gut (2003) 52:735–41. 12692061 10.1136/gut.52.5.735 PMC1773650

[93] Peng L Li ZR Green RS Holzman IR Lin J. Butyrate enhances the intestinal barrier by facilitating tight junction assembly via activation of AMP–activated protein kinase in Caco−2 cell monolayers. J Nutr. (2009) 139:1619–25. 10.3945/jn.109.104638 19625695 PMC2728689

[94] Kim M Kim CH. Regulation of humoral immunity by gut microbial products. Gut Microbes (2017) 8:392–9. 10.1080/19490976.2017.1299311 28332901 PMC5570419

[95] Kasubuchi M Hasegawa S Hiramatsu T Ichimura A Kimura I. Dietary gut microbial metabolites, short–chain fatty acids, and host metabolic regulation. Nutrients (2015) 7:2839–49. 10.3390/nu7042839 25875123 PMC4425176

[96] Ohira H Fujioka Y Katagiri C Mamoto R Aoyama–Ishikawa M Amako K. Butyrate attenuates inflammation and lipolysis generated by the interaction of adipocytes and macrophages. J Atheroscler Thromb. (2013) 20:425–42. 23470566 10.5551/jat.15065

[97] Nastasi C Candela M Bonefeld CM Geisler C Hansen M Krejsgaard T. The effect of short–chain fatty acids on human monocyte–derived dendritic cells. Sci Rep. (2015) 5:16148. 10.1038/srep16148 26541096 PMC4635422

[98] Fontenelle B Gilbert KM n–Butyrate anergized effector CD4^+^T cell generation or activity. Scand J Immunol. (2012) 76:457–63. 10.1111/j.1365-3083.2012.02740.x 22724664

[99] Brown AJ Goldsworthy SM Barnes AA Eilert MM Tcheang L Daniels D. The Orphan G protein–coupled receptors GPR41 and GPR43 are activated by propionate and other short chain carboxylic acids. J Biol Chem. (2003) 278:312–9. 10.1074/jbc.M211609200 12496283

[100] Nilsson NE Kotarsky K Owman C Olde B. Identification of a free fatty acid receptor, FFA2R, expressed on leukocytes and activated by short–chain fatty acids. Biochem Biophys Res Commun. (2003) 303:1047–52. 12684041 10.1016/s0006-291x(03)00488-1

[101] Thangaraju M Cresci GA Liu K Ananth S Gnanaprakasam JP Browning DD. GPR109A is a G–protein–coupled receptor for the bacterial fermentation product butyrate and functions as a tumor suppressor in colon. Cancer Res. (2009) 69:2826–32. 10.1158/0008-5472.CAN-08-4466 19276343 PMC3747834

[102] Pluznick JL Protzko RJ Gevorgyan H Peterlin Z Sipos A Han J. Olfactory receptor responding to gut microbiota– derived signals plays a role in renin secretion and blood pressure regulation. Proc Natl Acad Sci USA. (2013) 110:4410–5. 10.1073/pnas.1215927110 23401498 PMC3600440

[103] Kim MH Kang SG Park JH Yanagisawa M Kim CH. Short–chain fatty acids activate GPR41 and GPR43 on intestinal epithelial cells to promote inflammatory responses in mice. Gastroenterology (2013) 145:e1–10. 10.1053/j.gastro.2013.04.056 23665276

[104] Le Poul E Loison C Struyf S Springael JY Lannoy V Decobecq ME. Functional characterization of human receptors for short chain fatty acids and their role in polymorphonuclear cell activation. J Biol Chem. (2003) 278:481–9. 10.1074/jbc.M301403200 12711604

[105] Park J Kim M Kang SG Jannasch AH Cooper B Patterson J. Short–chain fatty acids induce both effector and regulatory T cells by suppression of histone deacetylases and regulation of the mTOR–S6K pathway. Mucosal Immunol. (2015) 8:80–93. 10.1038/mi.2014.44 24917457 PMC4263689

[106] Nakajima A Nakatani A Hasegawa S Irie J Ozawa K Tsujimoto G. The short chain fatty acid receptor GPR43 regulates inflammatory signals in adipose tissue M2– type macrophages. PLoS ONE (2017) 12:e0179696. 10.1371/journal.pone.0179696 28692672 PMC5503175

[107] Arpaia N Campbell C Fan X Dikiy S van der Veeken J deRoos P. Metabolites produced by commensal bacteria promote peripheral regulatory T–cell generation. Nature 504:451–5. 10.1038/nature12726 24226773 PMC3869884

[108] Goverse G Molenaar R Macia L Tan J Erkelens MN Konijn T Diet–derived short chain fatty acids stimulate intestinal epithelial cells to induce mucosal tolerogenic dendritic cells. J Immunol. (2017) 198:2172–81. 10.4049/jimmunol.1600165 28100682

[109] Kim M Qie Y Park J Kim CH. Gut microbial metabolites fuel host antibody responses. Cell Host Microbe (2016) 20:202–14. 10.1016/j.chom.2016.07.001 27476413 PMC4982788

[110] Furusawa Y Obata Y Fukuda S Endo TA Nakato G Takahashi D. Commensal microbe–derived butyrate induces the differentiation of colonic regulatory T cells. Nature (2013) 504:446–50. 10.1038/nature12721 24226770

[111] Tao R de Zoeten EF Ozkaynak E Chen C Wang L Porrett PM. Deacetylase inhibition promotes the generation and function of regulatory T cells. Nat Med. (2007) 13:1299–307. 10.1038/nm1652 17922010

[112] Sandin A Bråbäck L Norin E Björkstén B. Faecal short chain fatty acid pattern and allergy in early childhood. Acta Paediatr. (2009) 98:823–7. 10.1111/j.1651-2227.2008.01215.x 19173682

[113] Di Costanzo M Paparo L Aitoro R Cosenza L Nocerino R Cozzolino T Potential Beneficial Effects of Butyrate against Food Allergy. In: Cong–Jun Li, editors. Butyrate: Food Sources, Functions and Health Benefits. New York, NY: Biochemistry Research Trends (2014). p. 81–90.

[114] Aitoro R Paparo L Amoroso A Di Costanzo M Cosenza L Granata V. Gut microbiota as a target for preventive and therapeutic intervention against food allergy. Nutrients (2017) 9:E672. 10.3390/nu9070672 28657607 PMC5537787

[115] Nowak–Wegrzyn A Chatchatee P. Mechanisms of tolerance induction. Ann Nutr Metab. (2017) 70:7–24. 10.1159/000457915 28521317

[116] Wang H Ji Y Wu G Sun K Sun Y Li W. l–tryptophan activates mammalian target of rapamycin and enhances expression of tight junction proteins in intestinal porcine epithelial cells. J Nutr. (2015) 145:1156–62. 10.3945/jn.114.209817 25878205

[117] Hashimoto T Perlot T Rehman A Trichereau J Ishiguro H Paolino M. ACE2 links amino acid malnutrition to microbial ecology and intestinal inflammation. Nature (2012) 487:477–81. 10.1038/nature11228 22837003 PMC7095315

[118] Bessede A Gargaro M Pallotta MT Matino D Servillo G Brunacci C Aryl hydrocarbon receptor control of a disease tolerance defense pathway. Nature (2014) 511:184–90. 10.1038/nature13323 24930766 PMC4098076

[119] Pilotte L Larrieu P Stroobant V Colau D Dolusic E Frédérick R. Reversal of tumoral immune resistance by inhibition of tryptophan 2,3– dioxygenase. Proc Natl Acad Sci USA (2012) 109:2497–502. 10.1073/pnas.1113873109 22308364 PMC3289319

[120] Fallarino F Grohmann U Vacca C Bianchi R Orabona C Spreca A. T cell apoptosis by tryptophan catabolism. Cell Death Differ. (2002) 9:1069–77. 10.1038/sj.cdd.4401073 12232795

[121] Spits H Artis D Colonna M Diefenbach A Di Santo JP Eberl G. Innate lymphoid cells – a proposal for uniform nomenclature. Nat Rev Immunol. (2013) 13:145–9. 10.1038/nri3365 23348417

[122] Lanis JM Alexeev EE Curtis VF Kitzenberg DA Kao DJ Battista KD. Tryptophan metabolite activation of the aryl hydrocarbon receptor regulates IL−10 receptor expression on intestinal epithelia. Mucosal Immunol. (2017) 10:1133–44. 10.1038/mi.2016.133 28098246 PMC5515702

[123] Venkatesh M Mukherjee S Wang H Li H Sun K Benechet AP. Symbiotic bacterial metabolites regulate gastrointestinal barrier function via the xenobiotic sensor PXR and toll–like receptor 4. Immunity (2014) 41:296–310. 10.1016/j.immuni.2014.06.014 25065623 PMC4142105

[124] Hammerschmidt–Kamper C Biljes D Merches K Steiner I Daldrup T Bol–Schoenmakers M. Indole−3–carbinol, a plant nutrient and AhR–Ligand precursor, supports oral tolerance against OVA and improves peanut allergy symptoms in mice. PLoS ONE (2017) 12:e0180321. 10.1371/journal.pone.0180321 28666018 PMC5493375

[125] Hill C Guarner F Reid G Gibson GR Merenstein DJ Pot B. The International scientific association for probiotics and prebiotics consensus statement on the scope and appropriate use of the term probiotics. Nat Rev Gastro Hepat. (2014) 11:506–14. 10.1038/nrgastro.2014.66 24912386

[126] Sudo N Sawamura S Tanaka K Aiba Y Kubo C Koga Y. The requirement of intestinal bacterial flora for the development of an IgE production system fully susceptible to oral tolerance induction. J Immunol. (1997) 159:1739–45. 9257835

[127] Isolauri E Arvola T Sütas Y Moilanen E Salminen S. Probiotics in the management of atopic eczema. Clin Exp Allergy (2000) 30:1604–10. 10.1046/j.1365-2222.2000.00943.x 11069570

[128] Malin M Verronen P Korhonen H Syväoja EL Salminen S Mykkänen H. Dietary therapy with Lactobacillus GG, bovine colostrum or bovine immune colostrum in patients with juvenile chronic arthritis: evaluation of effect on gut defense mechanism. Inflammopharmacology (1997) 5:219–36. 17638132 10.1007/s10787-997-0001-1

[129] Kaila M Isolauri E Soppi E Virtanen E Laine S Arvilommi H. Enhancement of the circulating antibody secreting cellresponse in human diarrhea by a human Lactobacillus strain. Pediatr Res. (1992) 32:141–4. 1324462 10.1203/00006450-199208000-00002

[130] Hardy H Harris J Lyon E Beal J Foey AD. Probiotics, Prebiotics and Immunomodulation of gutmucosal defences: homeostatis and Immunopathology. Nutrients (2013) 5:1869–12. 10.3390/nu5061869 23760057 PMC3725482

[131] Kim JY Choi YO Ji GE Effect of oral probiotics (Bifidobacteriumlactis AD011 andLactobacillus acidophilus AD031) administration on ovalbumin–induced food allergy mouse model. J Microbiol Biotechnol. (2008) 18:1393–400. 18756099

[132] Torii A Torii S Fujiwara S Tanaka H Inagaki N Nagai H Lactobacillus acidophilus strain L−92 regulates theproduction of Th1 cytokine as well as Th2 cytokines. Allergol Int. (2007) 56:293–301. 10.2332/allergolint.O-06-459 17646735

[133] Niers LE Timmerman HM Rijkers GT van Bleek GM van Uden NO Knol EF. Identification of strong interleukin−10 inducinglactic acid bacteria which down–regulate T helper type 2 cytokines. Clin Exp Allergy (2005) 35:1481–89. 10.1111/j.1365-2222.2005.02375.x 16297146

[134] Takahashi N Kitazawa H Iwabuchi N Xiao JZ Miyaji K Iwatsuki K. Oral administration of an immunostimulatoryDNA sequence from Bifidobacterium longum improves Th1/Th2 balance in a murine model. Biosci. Biotechnol Biochem. (2006) 70:2013–7. 10.1271/bbb.60260 16926520

[135] Ghadimi D Helwig U Schrezenmeir J Heller KJ de Vrese M Epigenetic imprinting by commensal probiotics inhibitis the IL−23/IL−17 axis in a vitro model of the intestinal mucosal immune system. J Leukoc Biol. (2012) 92:895–911. 10.1189/jlb.0611286 22730546

[136] Maassen CB van Holten–Neelen C Balk F den Bak–Glashouwer MJ Leer RJ Laman JD. Strain–dependent induction of cytokine profiles in the gut by orally administered Lactobacillus strains. Vaccine (2000) 18:2613–23. 10.1016/S0264-410X(99)00378-3 10775795

[137] Smits HH Engering A van der Kleij D de Jong EC Schipper K van Capel TM. Selective probiotic bacteria induce IL−10– producing regulatory T cells *in vitro* by modulating dendritic cell function through dendritic cell– specific intercellular adhesion molecule 3–grabbing nonintegrin. J Allergy Clin Immunol. (2005) 115:1260–7. 10.1016/j.jaci.2005.03.036 15940144

[138] Cross ML Gill HS. Can immunoregulatory lactic acid bacteria be used as dietary supplements to limit allergies? Int Arch Allergy Immunol. (2001) 125:112–9. 10.1159/000053804 11435727

[139] Aitoro R Simeoli R Amoroso A Paparo L Nocerino R Pirozzi C Extensively hydrolyzed casein formula alone or with L. rhamnosus GG reduces β-lactoglobulin sensitization in mice. Pediatr Allergy Immunol. (2017) 28:230–7. 10.1111/pai.12687 27992668

[140] Hol J van Leer EH ElinkSchuurman BE de Ruiter LF Samsom JN Hop W The acquisition of tolerance towards cow's milk through probiotic supplementation: a randomized controlled trial. J Allergy Clin Immunol. (2008) 121:1448–54. 10.1016/j.jaci.2008.03.018 18436293

[141] Flinterman AE Knol EF Van Ieperen AG Timmerman HM Knulst AC Bruijnzeel-Koomen CAFM. Probiotics have a different immunomodulatory potential in vitro versus ex vivo upon oral administration in children with food allergy. Int Arch Allergy Immunol. (2007) 143:237–44. 10.1159/000099467 17290150

[142] Braat H van den Brande J van Tol E Hommes D Peppelenbosch M van Deventer S. *Lactobacillus rhamnosus* induces peripheral hyporesponsiveness in stimulated CD4^+^ T cells via modulation of dendritic cell function. Am J Clan Nutr. (2004) 80:1618–25. 10.1093/ajcn/80.6.1618 15585777

[143] Borthakur A Gill RK Tyagi S Koutsouris A Alrefai WA Hecht GA. The probiotic Lactobacillus acidophilus stimulates chloride/hydroxyl exchange activity in human intestinal epithelial cells. J Nutr. (2008) 138:1355–9. 10.1093/jn/138.7.1355 18567760 PMC2705118

[144] Borchers AT Keen CL Gershwin ME. The influence of yogurt/Lactobacillus on the innate and acquired immune response. Clin Rev Allergy Immunol. (2009) 22:207–30. 10.1007/s12016-002-0009-7 12043382

[145] Fu L Peng J Zhao S Zhang Y Su X Wang Y. Lactic acid bacteria-specific induction of CD4^+^Foxp3^+^T cells ameliorates shrimp tropomyosin induced allergic response in mice via suppression of mTOR signaling. Sci Rep. (2017) 7:1987. 10.1038/s41598-017-02260-8 28512288 PMC5434066

[146] Berni Canani R Paparo L Nocerino R Cosenza L Pezzella V Di Costanzo M Differences in DNA methylation profile of Th1 and Th2 cytokine genes are associated with tolerance acquisition in children with IgE–mediated cow's milk allergy. Clin Epigenetics (2015) 7:38 10.1186/2Fs13148-015-0070-8 25859290 PMC4391731

[147] Paparo L Nocerino R Cosenza L Aitoro R D'Argenio V Del Monaco V. Epigenetic features of FoxP3 in children with cow's milk allergy. Clin Epigenetics (2016) 8:86. 10.1186/s13148-016-0252-z 27525046 PMC4981981

[148] Karlsson H Larsson P Wold AE Rudin A. Pattern of cytokine responses to Gram–positive and Gram–Negative commensal bacteria is profoundly changed when monocytes differentiate into dendritic cells. Infect Immun. (2004) 72:2671–8. 10.1128/IAI.72.5.2671-2678.2004 15102775 PMC387913

[149] Mohamadzadeh M Olson S Kalina WV Ruthel G Demmin GL Warfield KL. Lactobacilli activate human dendritic cells that skew T cells toward T helper 1 polarization. Proc Natl Acad Sci USA. (2005) 102:2880–5. 10.1073/pnas.0500098102 15710900 PMC549474

[150] Zhang J Su H Li Q Wu H Liu M Huang J. Oral administration of Clostridium butyricum CGMCC0313.1 inhibits β-lactoglobulin–induced intestinal anaphylaxis in a mouse model of food allergy. Gut Pathog. (2017) 9:11. 10.1186/s13099-017-0160-6 28250847 PMC5322677

[151] Yang B Xiao L Liu S Liu X Luo Y Ji Q. Exploration of the effect of probiotics supplementation on intestinal microbiota of food allergic mice. Am J Translat Res. (2017) 9:376–85. 28337267 PMC5340674

[152] Maiga MA Morin S Bernard H Rabot S Adel–Patient K Hazebrouck S. Neonatal mono–colonization of germ–free mice with Lactobacillus casei enhances casein immunogenicity after oral sensitization to cow's milk. Mol Nutr Food Res. (2017) 61. 10.1002/mnfr.201600862 28318108

[153] Liu MY Yang ZY Dai WK Huang JQ Li YH Zhang J Protective effect of Bifidobacterium infantis CGMCC313–2 on ovalbumin–induced airway asthma and b–lactoglobulin induced intestinal food allergy mouse models. World J Gastroenterol. (2017) 23:2149–58. 10.3748/wjg.v23.i12.2149 28405142 PMC5374126

[154] Schiavi E Barletta B Butteroni C Corinti S Boirivant M Di Felice G. Oral therapeutic administration of a probiotic mixture suppresses established Th2 responses and systemic anaphylaxis in a murine model of food allergy. Allergy (2011) 66:499–508. 10.1111/j.1398-9995.2010.02501.x 21058959

[155] Thang CL Baurhoo B Boye JI Simpson BK Zhao X. Effects of *Lactobacillus rhamnosus* GG supplementation on cow's milk allergy in a mouse model. Allergy Asthma Clin Immunol. (2011) 7:20. 10.1186/1710-1492-7-20 22145744 PMC3261804

[156] Berni Canani R Nocerino R Terrin G Coruzzo A Cosenza L Leone L. Effect of Lactobacillus GG on tolerance acquisition in infants with cow's milk allergy a randomized trial. J Allergy ClinImmunol. (2012) 129:580–2. 10.1016/j.jaci.2011.10.004 22078573

[157] Berni Canani R Nocerino R Terrin G Frediani T Lucarelli S Cosenza L Formula selection for management of children with cow milk allergy influences the rate of acquisition of tolerance: a prospective multicenter study. J Pediatric. (2013) 163:771–7. 10.1016/j.jpeds.2013.03.008 23582142

[158] Berni Canani R Di Costanzo M Bedogni G Amoroso A Cosenza L Di Scala C Extensively hydrolyzed casein formula containing Lactobacillus rhamnosus GG reduces the occurrence of other allergic manifestation sin children with cow's milk allergy: 3–year randomized controlled trial. J Allergy Clin Immunol. (2017) 139:1906–13. 10.1016/j.jaci.2016.10.050 28043872

[159] Mammas IN Greenough A Theodoridou M Kramvis A Rusan M Melidou A Probiotics and allergy in infants –an update review. Pediatr Allergy Immunol. (2010) 21:e659–66. 10.1111/j.1399-3038.2010.01061.x 20659267

[160] Ghadimi D Fölster–Holst R de Vrese M Winkler P Heller KJ Schrezenmeir J. Effects of probiotic bacteria and their genomic DNA on TH1/TH2–cytokine production by peripheral blood mononuclear cells (PBMCs) of healthy and allergic subjects. Immunobiology (2008) 213:677–92. 10.1016/j.imbio.2008.02.001 18950596

[161] Donato KA Gareau MG Wang YJ Lactobacillus rhamnosus GG attenuates interferon–γ and tumor–necrosis factor–α- induced barrier dysfunction and pro–inflammatory signaling. Microbiology (2010) 156:3288–97. 10.1099/mic.0.040139-0 20656777

[162] Mileti E Matteoli G Iliev ID Rescigno M Comparison of the immunomodulatory properties of three probiotics strains of Lactobacilli using complex culture systems: prediction for *in vivo* efficacy. PLoS ONE (2009) 4:e7056 10.1371/journal.pone.0007056 19756155 PMC2738944

[163] Baldassarre ME Laforgia N Fanelli M Laneve A Grosso R Lifschitz C. Lactobacillus GG improves recovery in infants with blood in the stools and presumptive allergic colitis compared with extensively hydrolyzed formula alone. J Pediatr. (2010) 156:397–401. 10.1016/j.jpeds.2009.09.012 19880141

[164] Rachid R Keet CA. Current status and unanswered questions for food allergy treatments. J Allergy Clin Immunol Pract. (2018) 6:377–382. 10.1016/j.jaip.2017.10.023 29162426

[165] Tang ML Ponsonby AL Orsini F Tey D Robinson M Su EL. Administration of a probiotic with peanut oral immunotherapy: a randomized trial. J Allergy Clin Immunol. (2015) 135:737–44. 10.1016/j.jaci.2014.11.034 25592987

